# The Fracture Modes of Biomimetic Borosilicate Glass Protective Composite

**DOI:** 10.3390/ma18040739

**Published:** 2025-02-07

**Authors:** Jun Sun, Chunxu Zhao, Jun Li, Hai Mei, Xiang Liu, Shilin Yan

**Affiliations:** 1 Hubei Key Laboratory of Theory and Application of Advanced Mechanics, Wuhan University of Technology, Wuhan 430070, China; sunj@whut.edu.cn (J.S.); 15684711371@163.com (C.Z.); jun_li@whut.edu.cn (J.L.); meihai3166@whut.edu.cn (H.M.); 2 School of Computer Science and Artificial Intelligence, Wuhan University of Technology, Wuhan 430070, China; 3 Department of Mechanics and Engineering Science, Wuhan University of Technology, Wuhan 430070, China

**Keywords:** biomimetic design, transparent composite, ballistic experiment, borosilicate glass, fracture mode

## Abstract

The biomimetic structures in nature, such as shells, turtles, and other scaly organisms, inspire the design of transparent protective composites for enhancing their anti-penetration performance. Here, we designed the borosilicate glass composites with nacreous and tortoiseshell structures and examined their mechanical properties and damage mechanisms under high-speed impact using ballistics experiments. The effects of arrangements and tablet size on the dynamic performance of borosilicate glass composites were also investigated. The results suggest that the biomimetic structure exhibits better impact performance than traditional composites with whole plate structure. Using the biomimetic structure, the average damage area is decreased by 57.6–66.5% and the average energy dissipation is increased around 5% for the transparent composites. Compared to the aligned arrangements, the staggered arrangement of tablets is more beneficial to the anti-penetration when the staggered point is positioned symmetrically. In addition, the tablet size also plays a significant role, where a small tablet can decrease the average damage area around 15.4–24.1% and increase the average energy dissipation up to 4.2%. Therefore, the tortoiseshell structure with the staggered arrangement of small tablets is an optimal combination of the design parameters, which exhibits the best ballistic performance among other configurations due to the substantial enhancement of the locking effect at the tablet interface. This study provides valuable insights into the impact performance and fracture mode of the biomimetic structural composites, especially for the transparent armors of glass materials.

## 1. Introduction

Transparent protective structures have been extensively utilized in buildings and vehicles due to their good optical clarity and resistance performance [1,2,3]. The traditional transparent protective structure requires a significant thickness of transparent materials to impede projectile penetration, making it bulky, heavy, and difficult to carry. With limited weight and thickness, traditional transparent protective structures have become impractical with weak, resistant performance. Therefore, it is necessary to enhance the performance of transparent structures to satisfy both lightweight and resistant requirements. Strengthening glass material and structural configuration can reduce the weight and thickness of the transparent protective structure effectively while maintaining or even improving its mechanical performance.

A transparent protective structure is a sandwich structure commonly comprising transparent ceramic and glass [4,5]. The traditional transparent protective structure employs float soda-lime glass as a front layer or interlayer. Recently, with the development of the synthesis processing techniques of glass material, many innovative impact-resistant lightweight glass materials, such as Magnesia–Alumina spinel [6], Sapphire [1], and borosilicate (‘Pyrex’) glass [7], have been employed as transparent protective layers. Among them, the density of the borosilicate glass is around 2.2–2.3 g/cm^3^, which is lower than that of the float soda-lime glass (2.5–2.6 g/cm^3^) and other transparent ceramics (3.2–6.0 g/cm^3^). Particularly, since areal density is a significant indicator of armor, smaller density often makes borosilicate glass one of the most promising ballistic protection materials [8]. In addition, borosilicate glass often exhibits [9,10] high hardness [11], good impact resistance [5], and exceptional thermal stability and chemical resistance [12], which has been widely used in many engineering applications, even in a radiation environment [13]. Generally, most studies on the borosilicate glass protective composite focused on the enhancement of its strength through doping compounds like oxide and ion exchange [14,15]. The effects of structural configuration on the impact behaviors of borosilicate glass protective composite have not been well understood [4].

Inspired from the tissue structures of shells [16], scyllarus [17], and other scaly organisms [18,19] in nature, composite structures characterized with bionic configurations are demonstrated to significantly enhance protective performance, similar to that of evolved scaly organisms [20,21]_._ Gu et al. [16] discovered that materials with a two-stage hierarchical structure inspired by conch shell exhibit higher impact resistance compared to those without such a biomimetic structure. Ginzburg et al. [17] found that incorporating the knuckle structure of mantis shrimp into composites of carbon fiber and enhanced polymer enhances damage tolerance and energy dissipation capabilities. Wang et al. [18] demonstrated that the interlocking seam architecture within the tortoiseshell-structured composite panel can mitigate crack concentration in impact zones [22]. Current bionic design principles derived from nacre [23], tortoiseshell [24], or topological self-locking [25] have also shown improvement in mechanical property for transparent protective structures. Notably, in terms of ballistic performance, impact resistance, and energy dissipation, these principles can enhance glass armor at the interface while maintaining a low areal density. Regarding the difficulties in material processing, the regular and simple shapes of tablets, such as hexahedral and octahedral, were used to mimic the nacreous and tortoiseshell biomimetic composites. In our previous studies, Hang Yuan et al. [26] and Xin Zhang et al. [27] proposed nacre-inspired and tortoiseshell-inspired double-layer float soda-lime glass panels in the transparent protective structure. Their anti-penetration performance was assessed through a ballistic impact test [4], employing a projectile that offers a more effective evaluation of the composites’ mechanical properties under high-speed impact conditions compared to drop tests [28,29]. The cracks propagation, energy dissipation, and depth of penetration were analyzed for evaluation. The results suggest that these two biomimetic structures significantly enhance the penetration resistance of glass panels. However, limited by the ballistic experimental technology [30], the damages for both biomimetic structural composites were lacking in being quantitively analyzed. The effects of the structural parameters on the fracture modes of brick-inspired and tortoiseshell-inspired glass composite structures, especially for borosilicate glass, still need to be further understood.

With the recent development of Artificial Intelligence (AI) technology, several AI models have been developed for the evaluation of ballistic performance [31,32] and fracture segmentation [33]. In the present study, the effects of structural parameters, including stacking configurations and the size of the tablet, on the impact behaviors and fracture mode of both brick-inspired and tortoiseshell-inspired borosilicate glass composites were examined through ballistic experiments and an AI-assisted fracture detection tool. The results suggest that the impact resistance of the tortoiseshell structure is better than that of the nacreous structure. The biomimetic glass plate with a staggered arrangement in a diagonal direction facilitates the anti-penetration. The mechanical strength tends to be improved when the overlapped zone between tablets is more significant, with smaller damage areas and higher rebound velocity.

## 2. Experiment Procedure

### 2.1. Bionic Configuration of Borosilicate Glass Layer

**Figure 1:**
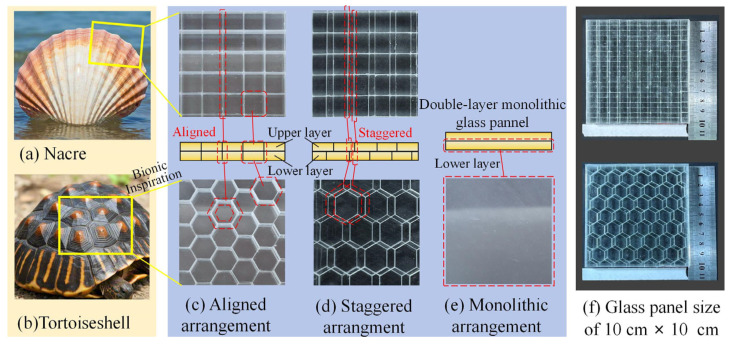
Different structures of double-layer glass panels and their dimensional size.

Borosilicate glass was adopted as both the front layer and interlayer in the transparent target plate, playing significant roles in impeding penetration [26]. A double-layer glass panel was utilized to fulfill these roles, consisting of hundreds of tablets arranged in a specific manner. Figure 1 illustrates the tablets’ arrangement, which mimics the three-dimensional hierarchical structures found in nature: the “brick-and-mortar” arrangement [23] of nacre from mollusk shells (Figure 1a) and the “rib–suture” arrangement [19] of the shell from a turtle carapace (Figure 1b). Each layer in the bionic structures comprised small borosilicate glass tablets, forming aligned (Figure 1c) or staggered (Figure 1d) arrangements of the borosilicate glass layers. The aligned (Figure 1c) or staggered (Figure 1d) arrangements of tablets were implemented with or without various offsets between the front layer and interlayer tablets. The traditional structure of a double-layer glass panel comprised two monolithic layers, as shown in Figure 1e.

The dimensions of the glass panel were 10 cm × 10 cm (Figure 1f), with a total thickness of 12 mm, comprising 10 mm of a double-layer glass panel and 2 mm of a back-side PMMA. In both biomimetic structural glass panels, each sublayer was assembled by hundreds of hexahedral or octahedral tablets with aligned or staggered arrangements. The details of the experimental samples are listed in Table 1.

**Figure 2:**
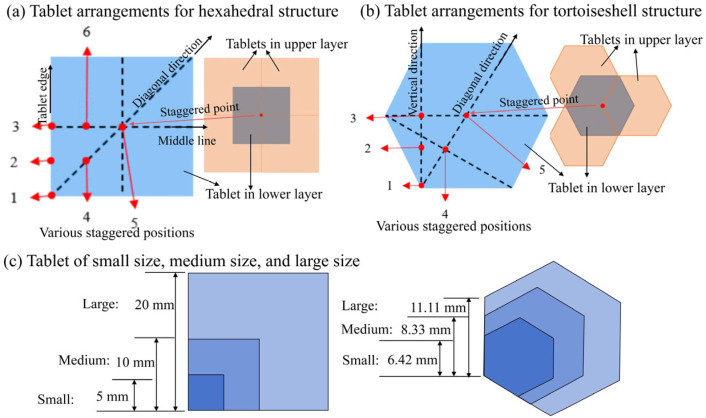
Various influence factors of structural parameters.

Figure 2 displays the structural parameters of the biomimetic structural glass panel with various configurations. The hexahedral tablets were employed to mimic the nacre-like (Figure 2a) structure, while the octahedral tablets were employed to mimic the tortoiseshell-like (Figure 2b) structure. The influence of tablet arrangement in the two layers was further analyzed in nacreous and tortoiseshell structures. Along with the aligned arrangement, six types of staggered tablet arrangements were implemented by paving the upper tablets in six positions relative to the lower tablets. Three directions were considered including in the vertical direction (or along the tablet edge), at the middle (or along the middle line), and in the diagonal direction. Three typical positions in each direction included the starting point (at the corner of tablet), the ending point (at the center of tablet), and their middle point. To further consider the effect from the tablet size, smaller tablet sizes of 5 mm and 6.42 mm were employed for the hexahedral and octahedral structural tablets. And larger tablet sizes of 20 mm and 11.11 mm were employed for them. The small sizes were carefully designed to be adequately large for the projectile to impact upon a single tablet with small boundary effects. Therefore, there were seventeen groups of structural configurations, and three tests were implemented for each group. In addition, we also performed ballistic experiments on the monolithic glass-based (Figure 1e) protective structure to compare with the biomimetic structures.

### 2.2. Material Processing of Biomimetic Structure

**Figure 3:**
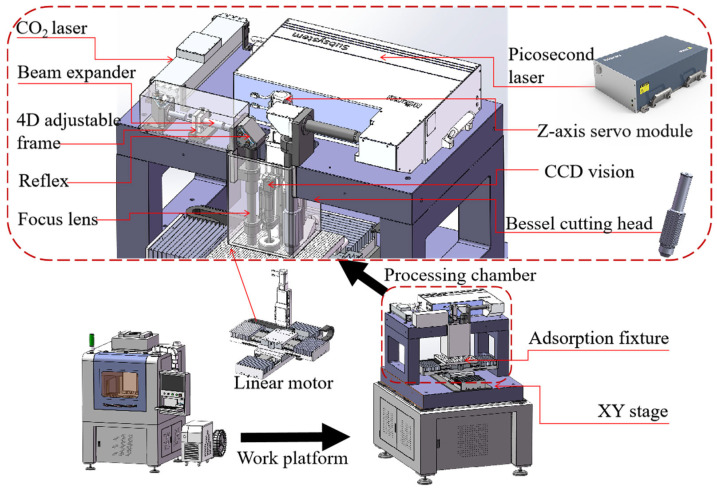
Schematic drawing of machining apparatus.

Initially, the biomimetic borosilicate glass layer was monolithic (Figure 1e). This monolithic layer was subsequently sliced into small tablets using an integrated glass cutting and fragmentation machine (Figure 3). The material processing primarily involved two steps: cutting and fragmenting. The raw material for the monolithic borosilicate glass layer was placed on a fixture-equipped platform. A laser beam emitted from an infrared picosecond laser was used for preliminary cutting. During this process, the laser power, cutting speed, and focal length were adjusted to achieve a glass blank that closely matched the desired thickness and shape. Subsequently, a CO_2_ laser was employed for the fine segmentation of the preliminary glass blank, ensuring dimensional accuracy and further refining the hexahedral or octahedral shape.

During the machining process, the position of the laser beam was adjusted through the *Z*-axis servo module. Finally, various shapes of glass tablets were produced and assembled into biomimetic structures like nacre and tortoiseshell. It should be noted that the laser system comprised a Bessel cutting head, a 4D adjustable frame, reflex, a focus lens, and CCD vision in addition to the lasers, and so on. The detailed information of the machining apparatus is listed in Table 2.

### 2.3. Ballistic Impact Test

**Figure 4:**
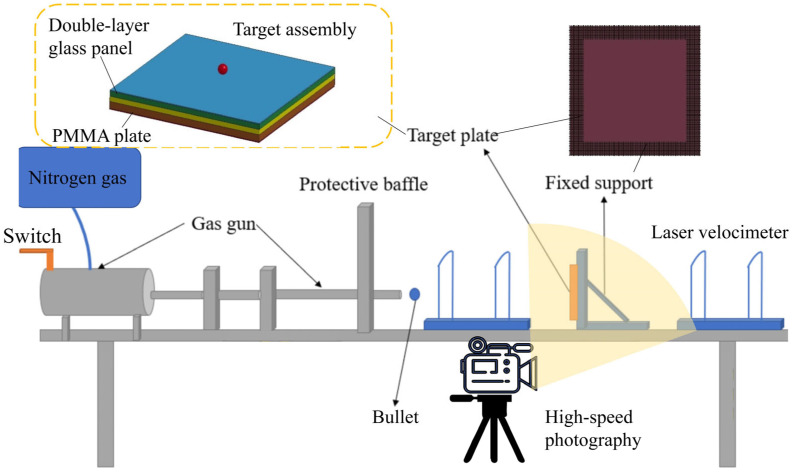
Schematic drawing of high-speed ballistic test system.

The experimental setup is depicted in Figure 4. The ballistic test system comprised a nitrogen cylinder, a small air gun, a projectile, a buffer system, a laser velocimetry system, a fixed support, and a high-speed photography system. The projectile was a spherical steel ball with a diameter of 5.86 mm and a mass of 0.8 g. The laser velocimetry system was utilized to measure both the incident and residual velocities, along with a Phantom v2012 high-speed camera. The camera was set to capture images at a time interval of 10 μs, achieving a frame rate of 100,000 frames per second, which allowed for clear images with a resolution of 768 × 256 pixels.

The target assembly comprised the double-layer borosilicate glass panel and a backing polymethyl methacrylate (PMMA [34]) layer. The glass panel was bonded to the backing PMMA layer using a Polyvinyl Butyral (PVB) membrane [35], which also enveloped the targe assembly. The tablets of both glass layers were arranged compactly together without any adhesive. Since borosilicate glass, PMMA, and PVB exhibit good transparency, it was easy to capture the specimen’s dynamic process and fracture characteristics accurately. The specimens were securely mounted in the fixed support using bolts.

**Figure 5:**
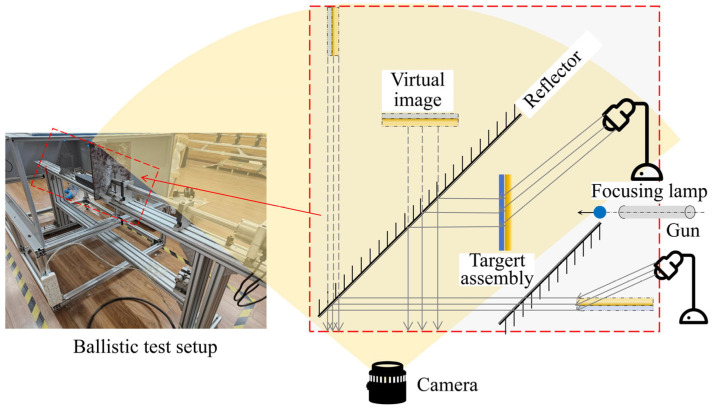
The employed experiment system.

Figure 5 illustrates the experimental setup in our laboratory. The ballistic experimental system was enclosed within a transparent shield to ensure safety and protection. In this system, the projectile’s incidence velocity was controlled by adjusting the gas pressure in the gas cylinder, which was closely set to 255 m/s. The target assembly was fixed 45 cm from the muzzle along the ballistic path. The high-speed camera was positioned on the side, with mirrors arranged specially to capture the simultaneous process in side views. Additionally, the position of the spotlights was optimized to provide brighter mirror images of the captured views.

### 2.4. Damage Dectection

The manual labeling of the glass panels’ fractures was conducted using the Supervisely tool [36], an online platform designed for image labeling and annotation. Given that the manual segmentation of complex shapes for post-test specimen fractures is extremely time-consuming, the AI-assisted detection tool in Supervisely (Estonia, Tallinn, Kesklinna linnaosa, Ahtri tn 12) has been proven to yield high-quality results at a lower cost in quantifying the fractures [33,37]. All images of the post-test specimens were captured at a resolution of 2000 pixels by 2000 pixels, with a pixel density of 20 pixels per millimeter.

**Figure 6:**
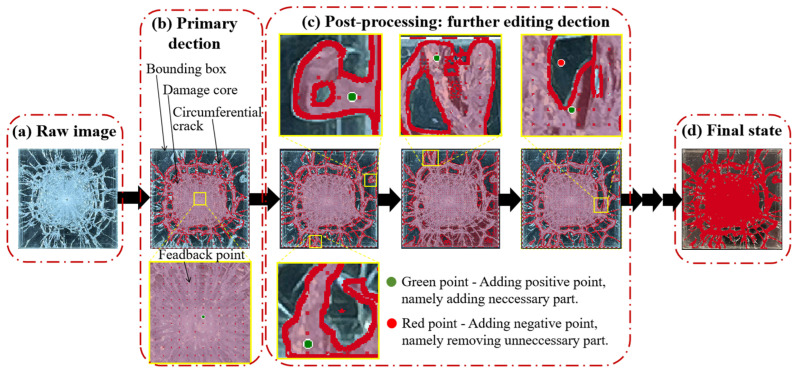
Damage detection process using the AI-assisted tool Supervisely.

After inputting the raw images (Figure 6a), the rough fractures corresponding to each experimental sample were automatically detected using bounding boxes (Figure 6b), facilitated by the interactive segmentation smart tool within Supervisely. Most primary features, such as the damage core, could be captured due to the opacity of the damaged sections within the glass layers. The intact or fractured state of the layers was subsequently refined through manual operations, including the addition of positive or negative feedback points (Figure 6d). Additionally, the brush, eraser, and pen tools were utilized to remove unnecessary parts or add new details in complex shapes. The final state of the damaged glass layer was labeled, and the corresponding area was also delineated.

## 3. Results and Discussions

### 3.1. Influence of Biomimetic Structures on the Dynamic Response of Transparent Protective Composite

**Figure 7:**
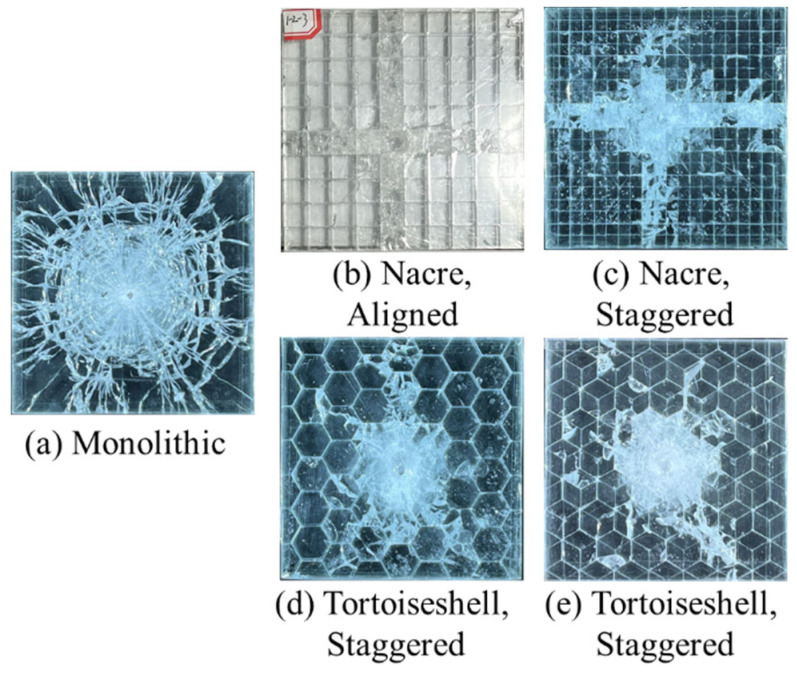
Damage patterns for various biomimetic structures. Glass panels of (**a**) Monolithic, (**b**) Nacreous aligned, (**c**) Nacreous staggered, (**d**) Tortoiseshell aligned, (**e**) Tortoiseshell staggered arrangements after impact.

Figure 7 displays the borosilicate glass samples with various biomimetic structures after impact. The damage pattern varies in the three structures. The entire target is completely broken for the monolithic structural sample, exhibiting circumferential cracks, radial cracks, a central material fragmentation zone, etc. The damage is a tensile failure, stemming from a lower stress threshold in the tensile crack propagation, and thus predominates the formation of the fracture surface [17]. Compared to the monolithic double-layer glass panel, the damage pattern is cross-shaped for nacreous structural samples with aligned and staggered arrangements (Figure 2b,c). Cracks and fractures are almost locked within the glass tablets along the cross-shaped fracture paths, while other glass tablets remain transparent and intact [16]. For tortoiseshell-structured samples, the damage zone caused by cracks is close to a circle at the center of the target, where cracks and fractures are locked within the glass tablets near the impact point. Notably, less discrete cracks and interface damages are observed in the tortoiseshell-structured composites compared to the nacreous glass panel with the hexahedral tablets.

**Figure 8:**
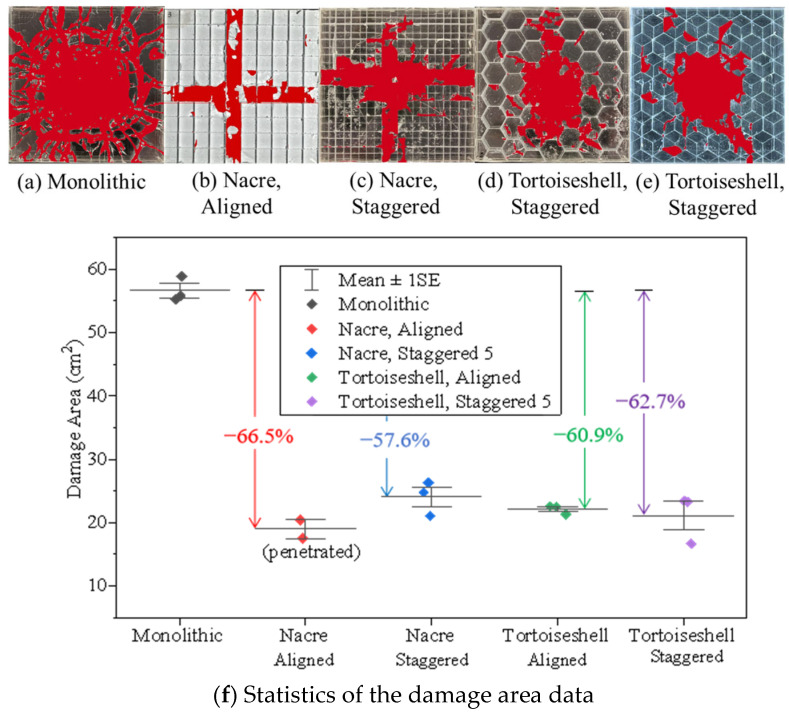
Damage areas for various biomimetic structures. Post-processing images of the (**a**) Monolithic, (**b**) Nacreous aligned, (**c**) Nacreous staggered, (**d**) Tortoiseshell aligned, (**e**) Tortoiseshell staggered glass panels after impact and (**f**) the corresponding damage area data.

Figure 8 displays the damage zone recognized using the Supervisely smart labeling tool. Here, we selected some glass tablets (Figure 7) with complex damaged features and calculated the damage areas for the fractured glass tablets (see Figures S1 and S2). It shows that the AI-recognized areas agree well with those of the experimentally observed damage zones for various biomimetic structures (Figure 8a–e). The statistical data suggest that the damage areas of the biomimetic structures range from 20 to 25 cm^2^, smaller than those of the traditional monolithic structure (Figure 8f) of approximately 56 cm^2^. The damage area of the double-layer glass panel was averagely decreased by 57.6–66.5% after being machined into the biomimetic structure. Similarly to the float soda-lime glass double-layer panel [26], those biomimetic structures of the borosilicate glass significantly reduce the damage area, improving protective performance compared to the traditional structure. The damage areas of the bionic structural composites decrease significantly because crack propagation is inhibited within the fractured tablets, leading to the complete dissipation of impact energy within these tablets. Subsequently, the nearby tablets take over the role of dissipating any residual energy, thereby indirectly enhancing the fracture threshold of the adjacent tablets through the bionic design. Compared to the tortoiseshell-structured composite, the damage areas of the nacre-structured ones are more significant. This is because the hexahedral tablet with fewer boundaries facilitates stress wave transmission along the cross-shaped paths. In contrast, the tortoiseshell tablets with more boundaries enhance the reflection and superposition of local stress waves, leading to the circularly centralized damage zone. The results suggest that the tortoiseshell structure performs better in impeding the projectile with a smaller damage area, due to the interlocking effect between the two borosilicate glass layers.

**Figure 9:**
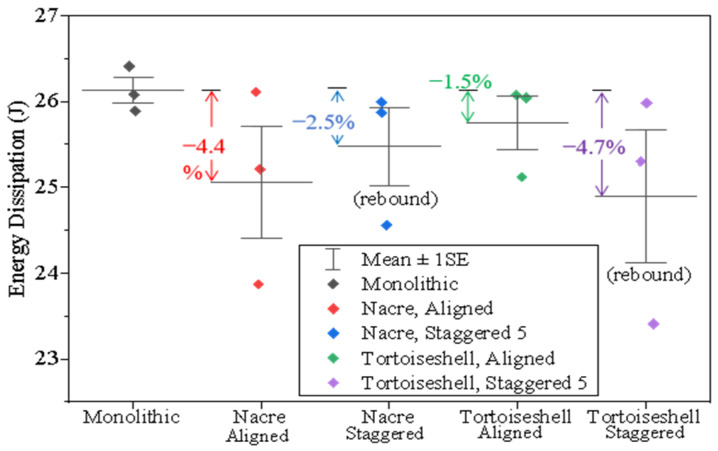
Statistics of energy dissipation for various biomimetic structures.

Figure 9 displays the energy dissipation for various biomimetic structures. The energy dissipation was calculated by subtracting the residual kinetic energy from the initial kinetic energy of the projectile, i.e., $\frac{1}{2}$ *m*_projectile_ (*V*^2^_intial_ − *V*^2^_residual_). The consumed impact energy of the borosilicate glass panel is 26.0 J/cm^3^, higher than that of the float soda-lime glass panel (25.3 J/cm^3^) [26]. This is because the borosilicate glass is more ductile [7,38]. The biomimetic structures consume less impact energy with smaller fracture areas than the monolithic structure. However, the two biomimetic structures have different relationships between the energy dissipation and the damage area. For the biomimetic structures with staggered configurations, the projectiles tend to rebound. According to the equation of $\frac{1}{2}$ *m*_projectile_ (*V*^2^_intial_ − *V*^2^_residual_), the average energy dissipation for the staggered configuration of the tortoiseshell structure is 4.7%, which is higher than that of the nacreous structure (2.5%). This indicates that the rebound velocity of the tortoiseshell structure, when impacted, is greater than that of the nacreous structure. Furthermore, the staggered tortoiseshell structure is stiffer than the staggered nacreous structure. This result effectively demonstrates that the tortoiseshell structure with staggered arrangements exhibits better impact resistance.

Consistent with our previous studies of biomimetic transparent composites using float soda-lime glass [26,27], cross-shaped cracks occurred in the nacreous structure, whereas circular cracks were observed in the tortoiseshell structure, regardless of the staggered arrangement of tablets. Furthermore, the fracture accumulated within the tablets in proximity to the impact point. These findings indicate that the biomimetic structures incorporated into the borosilicate glass panel effectively hinder crack propagation through the split tablets and contain stress waves within these tablets along the crack paths. Particularly, the interlocking action between tablets is augmented when fractured tablets and irregular fragmentation occur during impact. Since the fractures develop along the edges of the tablets, which serve to trap the impact energy, the biomimetic structure ultimately dictates the damage pattern of the transparent composites.

### 3.2. Influence of the Tablet Arrangement for the Nacre-Structured Composites

**Figure 10:**
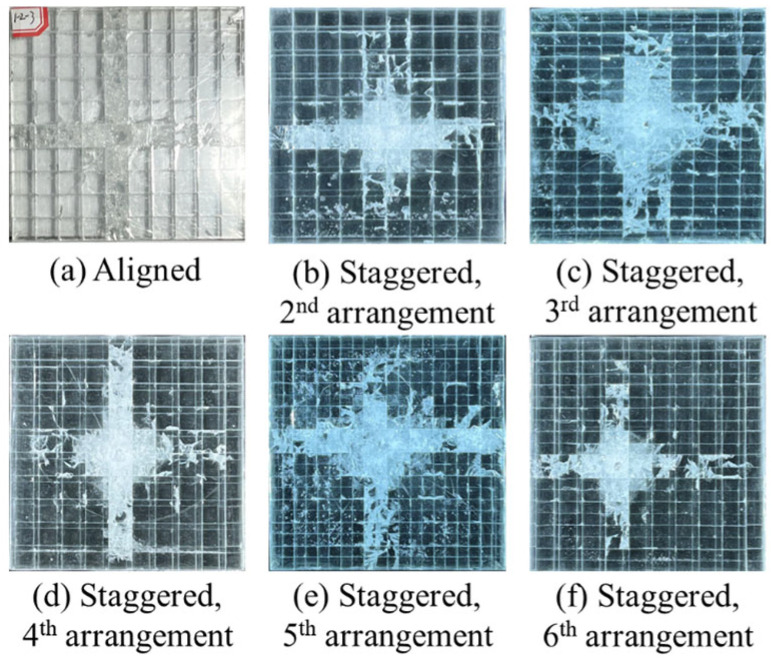
Damage patterns for various tablet arrangements of the nacreous structure. Glass panels of the (**a**) Aligned, (**b**) 2nd staggered, (**c**) 3rd staggered, (**d**) 4th staggered, (**e**) 5th staggered, (**f**) 6th staggered arrangments after impact.

To further understand the influence of the tablet arrangement, Figure 10 displays the damage pattern of the nacreous structural composites. The damaged zones are cross-shaped for all nacreous structural composites. After impact, the samples exhibit primary damage at the center and discrete interface damage in the remote region. This suggests that the fracture mode mainly depends upon the tablet’s shape or the nacreous structure. The tablet arrangements may influence the crack nucleation and propagation.

**Figure 11:**
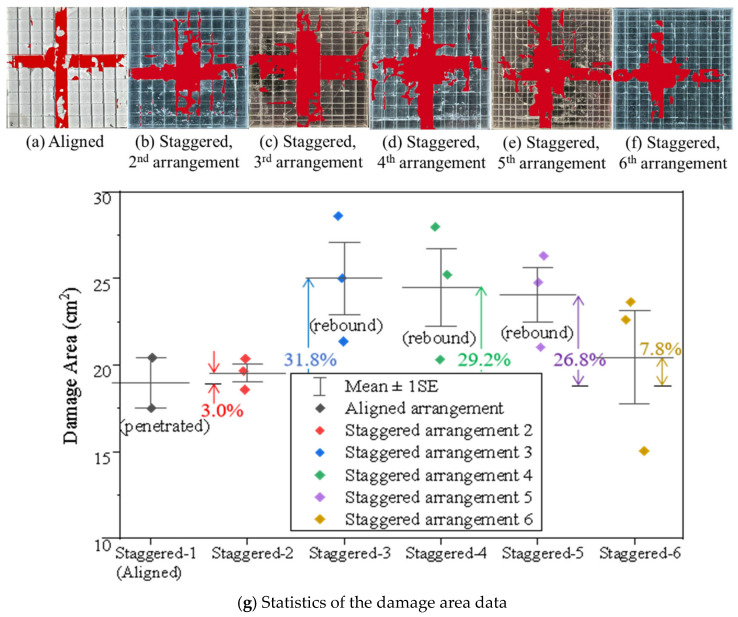
Damage areas for the nacreous transparent composites with various tablets’ arrangements. Post-processing images of the (**a**) Aligned, (**b**) 2nd staggered, (**c**) 3rd staggered, (**d**) 4th staggered, (**e**) 5th staggered, (**f**) 6th staggered glass panels after impact and (**g**) the corresponding damage area data.

Figure 11 displays the qualified damage zone for the nacre-structured composites using the AI-assisted labeling tool. When the staggered point moves from Position 1 to Position 3, the corresponding damage areas increase from 3.0% (Position 2) to 31.8% (Position 3), with the overlapping zone increasing along the composite’s edge (Figure 11g). However, the composite with the second staggered arrangement was penetrated (Figure 10a). In comparison, the projectile rebounded upon the composite with the third staggered arrangement (Figure 10c). To further consider the projectile rebounding, the staggered point moving in a diagonal direction (from Position 1 to Position 5 in Figure 2) was also analyzed, and the damage area for the fifth staggered arrangement is smaller than that of the third and fourth structures. Consequently, the staggered arrangement in the diagonal direction for the nacreous structure is more favorable in anti-penetration than in other directions.

**Figure 12:**
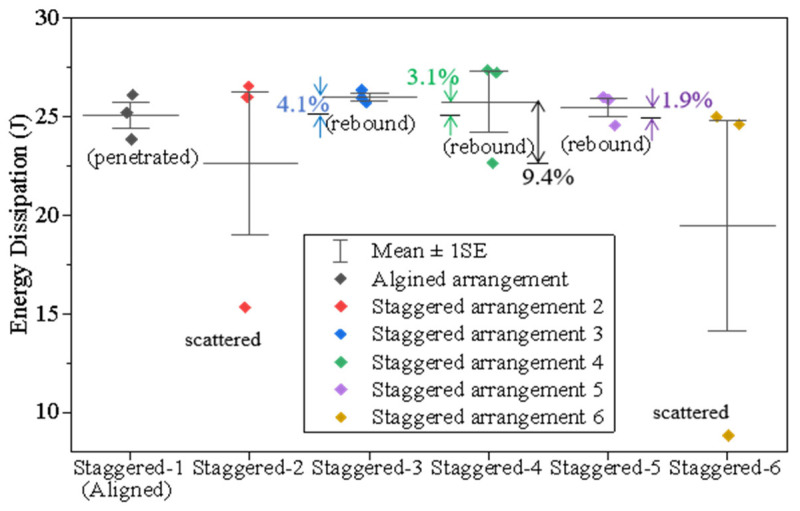
Statistics of energy dissipation for all nacreous structural composites.

Figure 12 displays the energy dissipations for the nacre-structured composites. Under high-speed impact, the nacreous composites with various tablets’ arrangements (see Figures S3 and S4) consume an average impact energy of 20–25 J. Compared to the damage areas, the energy dissipations for all staggered nacreous samples are closer to each other except the scattered data. Particularly, their discrepancies are no more than 10%. However, the average dissipated energies are lower when the overlapped zones between tablets increase in the diagonal direction (from Position 4 to Position 5) than in other directions. Particularly, the projectiles rebound upon the composites with those staggered arrangements. Combined with the damage area’s data, they suggest that the resistant performance of the nacreous structure is better when the staggered point is closer to the center of the tablet. It could also be found that the stiffness of the borosilicate glass composite is relatively high, therefore leading to a large rebound velocity after impact.

With various arrangements of staggered positions between the tablets of upper and lower layers, projectiles rebounded upon impacting the glass panels. This suggests that the partial or complete overlap zones were beneficial in protecting the glass panel against penetration. The staggered arrangement introduces normal resistance at the tablet interfaces, providing an additional mechanism for energy dissipation compared to the tangential shearing action observed in aligned glass panels. Furthermore, when the staggered position is symmetric or centered within the tablet, the normal resistance force is equally sustained by the tablets, further enhancing the stiffness of the biomimetic glass panel.

### 3.3. Influence of the Tablet Arrangement for the Tortoiseshell-Structured Composites

**Figure 13:**
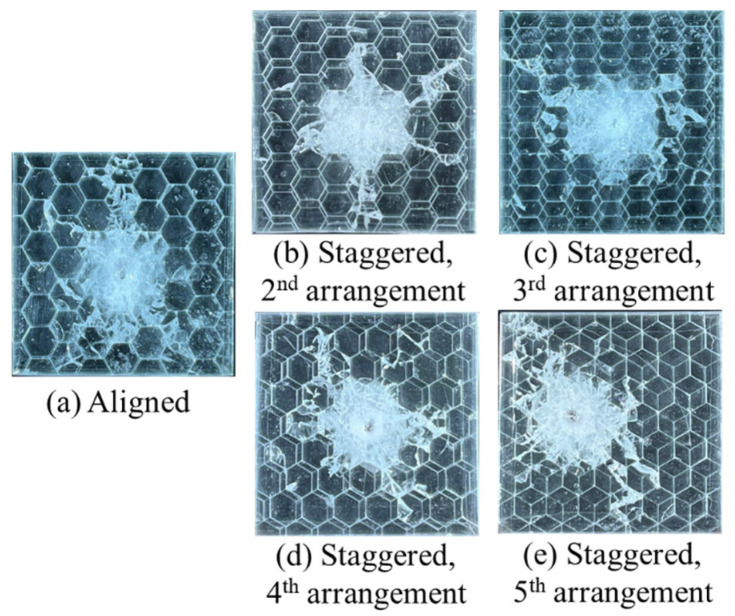
Damage patterns for the tortoiseshell structures. Glass panels of the (**a**) Aligned, (**b**) 2nd staggered, (**c**) 3rd staggered, (**d**) 4th staggered and (**e**) 5th staggered arrangements after impact.

Figure 13 displays the damage pattern for the tortoiseshell structural samples. The damage pattern is weakly related to the arrangement and mainly determined by the tablet’s shape or the tortoiseshell structure. The damaged zones are circular, and the nearby interface damages are distributed discretely along the edges of the tablets.

**Figure 14:**
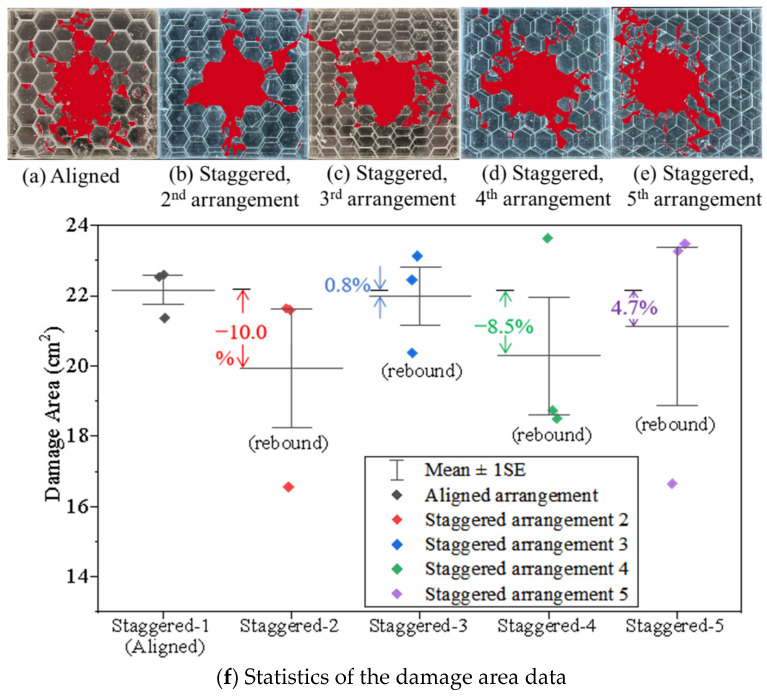
Damage areas for the tortoiseshell-structured composites with various tablets’ arrangements. Post-processing images of the (**a**) Aligned, (**b**) 2nd staggered, (**c**) 3rd staggered, (**d**) 4th staggered, (**e**) 5th staggered glass panels after impact and (**f**) the corresponding damage area data.

Figure 14 plots the damage areas recognized by AI technology for all tortoiseshell-structured composites with various arrangements. Their damage areas (Figure 14f) are lower than those of the nacreous structure (Figure 11g). Compared to the nacreous structural composite, the discrepancies in the damage area caused by variations in the staggered point are smaller (see Figures S5 and S6). Notably, the damage area is proportional to the overlapped zone between tablets in vertical and diagonal directions. Thus, the damage areas become larger (Figure 14f) when the staggered point moves from Position 2 to Position 3 or from Position 4 to Position 5 (Figure 2b).

**Figure 15:**
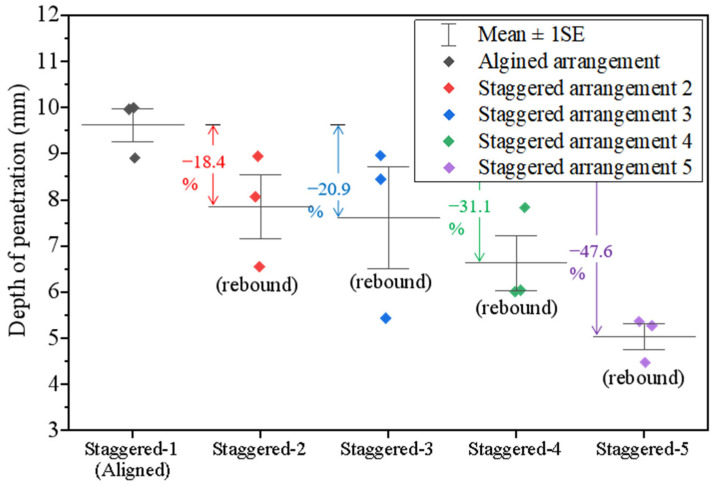
Depth of penetration for the tortoiseshell-structured composites with various tablet arrangements.

The depths of penetrations for the tortoiseshell structures with various arrangements are further measured and plotted in Figure 15. The tortoiseshell-structured composite is more difficult to be penetrated than the nacre-structured composite. In particular, staggered tablet arrangements often reduce the depth of penetration compared to the aligned arrangement. The depth of penetration is proportional to the staggered zone between tablets in both the vertical direction (from Position 2 to Position 3) and the diagonal direction (from Position 4 to Position 5). The results decrease from −18.4%, −20.9%, and −31.1% to −47.6% when the staggered point moves into the center of the composite. This suggests that the staggered arrangements in both directions for the tortoiseshell structure benefit from the anti-penetration performance. Based on the results from the nacreous structure, the fifth staggered tablet arrangement is the best for both biomimetic structures to resist penetration.

**Figure 16:**
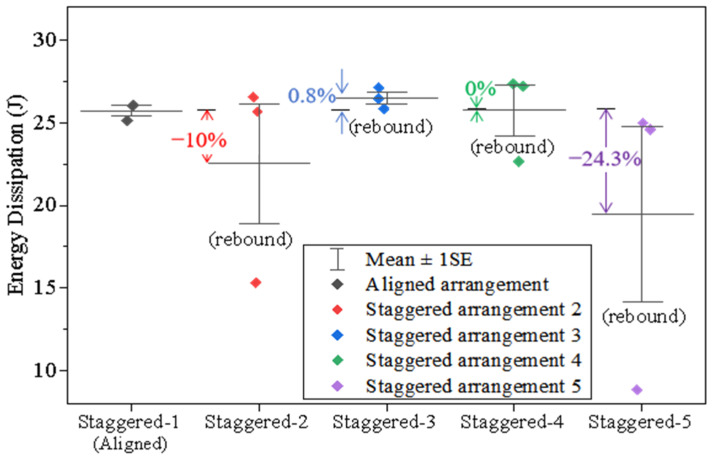
Statistics of energy dissipation for all tortoiseshell-structured composites.

Similarly to the nacreous structure, the average energy dissipation for all tortoiseshell structural samples ranges from 20 J to 25 J, as shown in Figure 16. However, the discrepancies of their dissipated energy are more significant with various staggered positions. Notably, the average energy dissipation decreases around 24.3% in the diagonal direction (from Position 1 to Position 5). It indicates that the composite using the fifth staggered arrangement is stiffer, since the corresponding rebound velocity is higher. In addition, the smaller depth of penetration, the staggered arrangement in the diagonal direction is more effective in impeding penetration and performs better for the tortoiseshell structure.

Using the staggered arrangement of tablets within the tortoiseshell-structured composites, the double-layer borosilicate glass panels exhibited resistance to penetration, causing the corresponding projectiles to rebound. Compared to the nacreous structure, the various staggered arrangements of the tortoiseshell structure had statistically insignificant effects on the shape and area of damage. This is attributed to the octahedral tablets having more edges than hexahedral tablets, which facilitates the interaction of stress waves within the tablets. Additionally, more squeezing and shearing actions contribute to energy dissipation during impact, further enhancing the locking effects at the tablet interfaces. Consequently, the influence of various staggered arrangements on the fracture mode of the tortoiseshell structure is insignificant.

### 3.4. Influence of the Tablet Thickness

**Figure 17:**
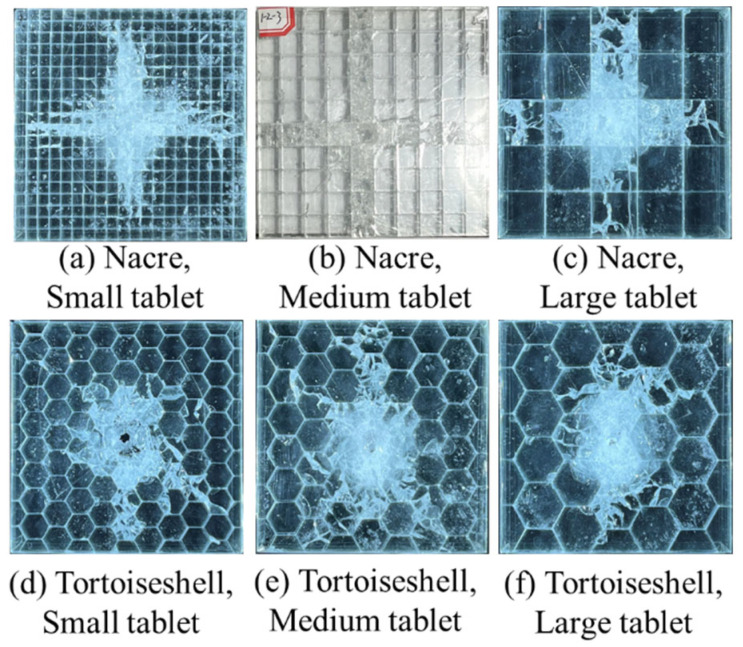
Damage patterns for biomimetic structures of various tablet sizes. Glass panels of the (**a**) small, (**b**) medium, (**c**) large tablets for the nacreous structure and the (**d**) small, (**e**) medium, (**f**) large tablets for the tortoiseshell structure after impact.

Figure 17 displays the damage pattern for the various tablet sizes of the aligned structural samples. Using the aligned arrangement, the patterns of double-layer glass panels with various tablet sizes are almost the same: cross-shaped for the nacreous structure (Figure 17a–c) and circular for the tortoiseshell structure (Figure 17d–f). The corresponding samples are penetrated in each group of various tablet sizes owing to the aligned arrangement. The results show that the tablet size has little effect on the damage pattern, although the damage zones of the glass plate with various tablet sizes are not the same.

**Figure 18:**
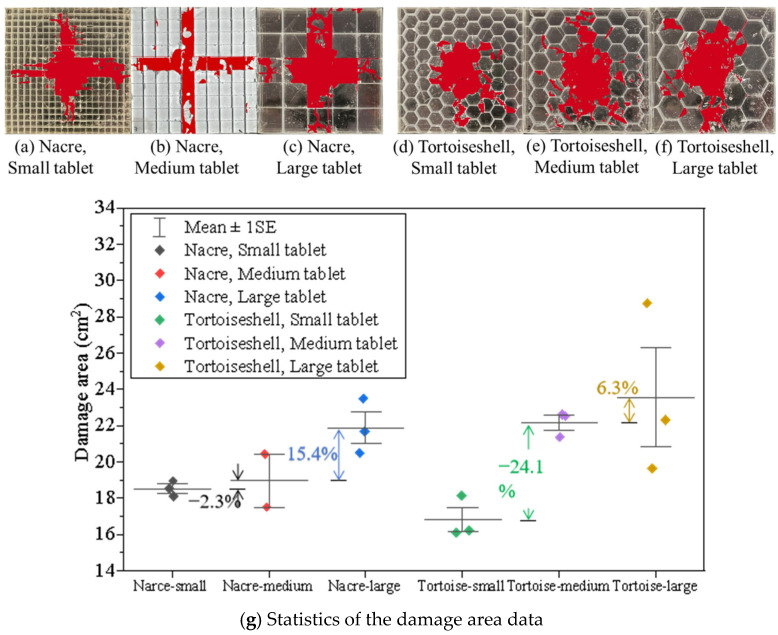
Damage areas for various sizes of biomimetic structures. Post-processing images of the nacreous structural glass panels with (**a**) small, (**b**) medium, (**c**) large tablets for and the tortoiseshell structure after impact with (**d**) small, (**e**) medium, (**f**) large tablets and (**g**) the corresponding damage area data.

Using the AI recognition technology, the damaged zones of the two biomimetic structural plates are plotted in Figure 18. The results show that the damage area is in proportion to the tablet’s size (Figure 18g). The damage area (see Figures S7 and S8) significantly increases from 17–18 cm^2^ to 24–27 cm^2^. For the nacreous structure of the medium-sized tablet, the average damage area decreases around 2.3% when the tablet size becomes smaller (Figure 18a) and increases around 15.4% when the tablet size becomes larger (Figure 18c). For the tortoiseshell structure of the medium-sized tablet, the average damage area decreases by 24.1% when the tablet size becomes smaller (Figure 18d) and increases around 15.4% when the tablet size becomes larger (Figure 18f). The result indicates that small tablets exhibit better performance in impeding the impact with smaller damage areas for both nacre- and tortoiseshell-structured composites. It could be explained that the composites comprising smaller tablets introduce more tablet edges, leading to more interfaces between tablets.

**Figure 19:**
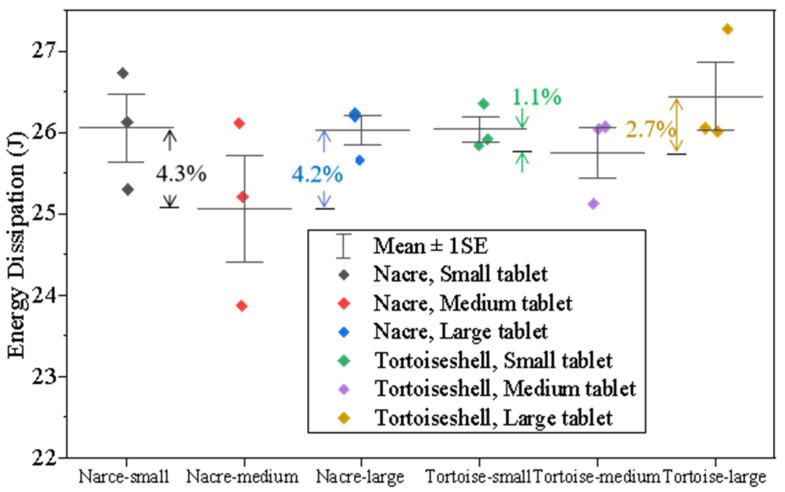
Energy dissipation for biomimetic structures of various tablet sizes.

Figure 19 displays the energy dissipation for the biomimetic structures with various tablet sizes. The results of three typical sizes for both nacreous and tortoiseshell structural glass plates show that the energy dissipations of both large and small glass tablets are more significant than those of the medium ones. Comparatively, the energy dissipations of the smaller tablets are higher than those of the larger ones. To consider both damage area and energy dissipation, therefore, a small-size tablet has the best capability of impeding the projectile for both nacreous and tortoiseshell structures. It suggests that a smaller tablet size is advantageous for promoting the interlocking effects in the biomimetic structure, therefore increasing the strength of the double-layer borosilicate glass panel.

Compared to various staggered arrangements, the influence of tablet size on damage characteristics and area is more significant. Specifically, tortoiseshell-structured borosilicate glass panels with smaller tablets have more tablet edges, resulting in smaller damage areas and greater energy dissipation compared to the nacreous structure. Additionally, smaller tablets have more edges engaging in stress wave interactions, leading to increased squeezing and shearing forces during energy dissipation. The locking effects at the tablet interfaces are also enhanced due to the increased number of irregularly fractured tablets. Consequently, tablet size is a more dominant factor than staggered arrangement in the impact behavior of these composites. Smaller tablets benefit the tortoiseshell structure by providing better resistance to penetration with a reduced damage area and increased energy dissipation.

## 4. Conclusions

In summary, this study examined the influences of biomimetic structures, specifically nacreous and tortoiseshell designs, on the fracture mode of double-layer glass protective composites using the ballistic impact method and an AI-assisted damage detection tool. Additionally, the effects of tablet arrangement and size were analyzed. The results indicate that the biomimetic structure is the most significant factor among the three considered. These findings are consistent with our previous research on float soda-lime glass, which demonstrated that biomimetic structural glass panels exhibit superior performance in impeding projectiles. When using the biomimetic structure, the average damage area decreased by 57.6% to 66.5%, and the average energy dissipation increased by approximately 5%. For both hexahedral and octahedral tablets, a staggered arrangement was more beneficial to the ballistic performance of the borosilicate glass panel than an aligned arrangement. Notably, the depth of penetration was significantly reduced for the tortoiseshell structure when the staggered point was positioned symmetrically or at the center, leading to increased stiffness in the biomimetic structure. In comparison to stacking configurations, tablet size had a more significant influence, with smaller tablets decreasing the average damage area by approximately 15.4% to 24.1% and increasing energy dissipation by up to 4.2%. Therefore, the tortoiseshell structure with the staggered arrangement of small tablets is an optimal combination of the design parameters, which exhibits the best ballistic performance among other configurations due to the substantial enhancement of the locking effect at the tablet interface.

This study provides valuable insights into the fracture behavior of biomimetic borosilicate glass panels and offers useful guidance for designing transparent composites with excellent impact resistance for applications in glass armor, bulletproof shields, and vehicle windshields. Although this work presents two typical biomimetic structures, further comparisons with other biomimetic designs, such as topologically interlocked structures, are needed to improve protective composite designs.

## Figures and Tables

**Table 1:** The details of experimental samples.

Group	Bionic Design	Tablet Shape	Configuration	Length (mm)	Tablet Arrangement
1	Traditional		Monolithic		
2	Nacre	Hexahedral	Aligned	10	1
3	Nacre	Hexahedral	Staggered	10	2
4	Nacre	Hexahedral	Staggered	10	3
5	Nacre	Hexahedral	Staggered	10	4
6	Nacre	Hexahedral	Staggered	10	5
7	Nacre	Hexahedral	Staggered	10	6
8	Nacre	Hexahedral	Aligned	5	1
9	Nacre	Hexahedral	Aligned	20	1
10	Tortoiseshell	Octahedral	Aligned	8.33	1
11	Tortoiseshell	Octahedral	Staggered	8.33	2
12	Tortoiseshell	Octahedral	Staggered	8.33	3
13	Tortoiseshell	Octahedral	Staggered	8.33	4
14	Tortoiseshell	Octahedral	Staggered	8.33	5
15	Tortoiseshell	Octahedral	Staggered	8.33	6
16	Tortoiseshell	Octahedral	Aligned	6.42	1
17	Tortoiseshell	Octahedral	Aligned	11.11	1

**Table 2:** The details of the machining apparatus.

No.	Apparatus	Function	Manufacturer	Instruction
1	Piscosecond laser	Cutting	Huaray, Wuhan, China	Model: PINE3-1064-60
2	CO_2_ laser	Fragmenting	Huaray, Wuhan, China	Power: 100 (W)
3	Cuting head	Cutting	EVENOPTICS, Shanghai, China	Type: Bessel
4	Focus lens	Optical focusing	Sintec, Wuhan, China	Size: Ø25 × 50.8 (mm)
5	Absorption fixture	Fixture	Homemade, Wuhan, China	Size: 350 × 350 (mm)
6	Linear motor	Dynamic module	TGB, Wuhan, China	Size: 400 × 400 × 100 (mm)
7	Chiller for laser	Cooling, feedback	TONGFEI, Hebei, China	Temperature: 20–35 (°C)

## Data Availability

All original data regarding where data supporting reported results can be found in the Supplementary Materials.

## Supplementary Materials

The following supporting information can be downloaded at: https://www.mdpi.com/article/10.3390/ma18040739/s1, Figure S1: Damage patterns for various biomimetic structures; Figure S2: Damage areas calculated by Artificial Intelligence for various biomimetic structures; Figure S3: Damage patterns for various tablet arrangements of the nacreous structure; Figure S4: Damage areas calculated by Artificial Intelligence for the nacreous transparent composites with various tablets’ arrangements; Figure S5: Damage patterns for the tortoiseshell structures; Figure S6: Damage areas calculated by Artificial Intelligence for the tortoiseshell-structured composites with various tablets’ arrangements; Figure S7: Damage patterns for biomimetic structures of various tablet sizes; Figure S8: Damage areas calculated by Artificial Intelligence for various sizes of biomimetic structures.
