# Supplementary material for: The Fracture Modes of Biomimetic Borosilicate Glass Protective Composite
Source: Materials (Basel). 2025 Feb 7;18(4):739. doi: 10.3390/ma18040739 (PMC11857408; doi:10.3390/ma18040739)
Supplement: Supplementary file 1 [file materials-18-00739-s001.zip › materials-3413831-supplementary.pdf]

# Supplementary Issue

# Figure

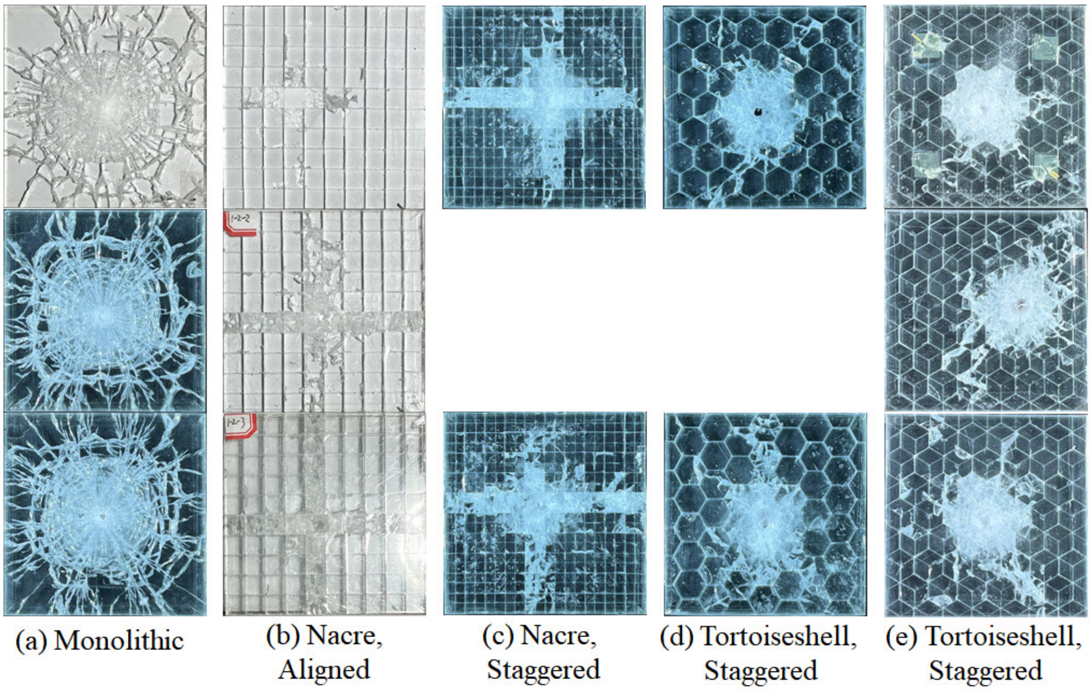


**Fig. S1. Damage patterns for various biomimetic structures**

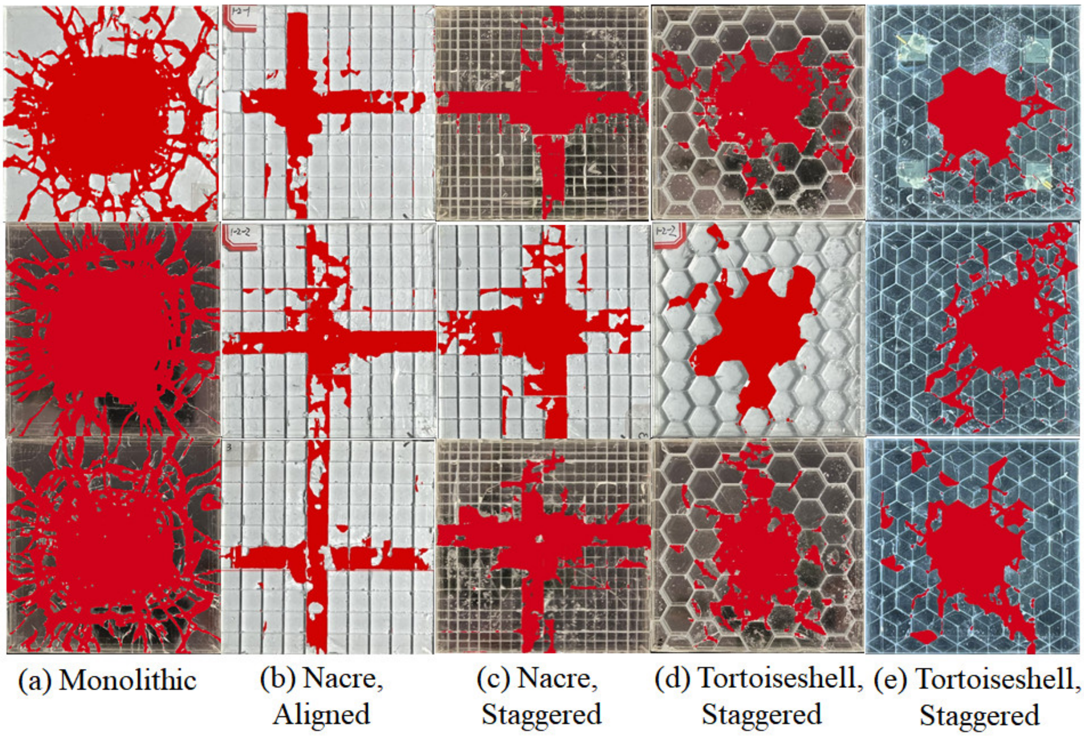


**Fig. S2 Damage areas calculated by Artificial Intelligence for various biomimetic structures.**

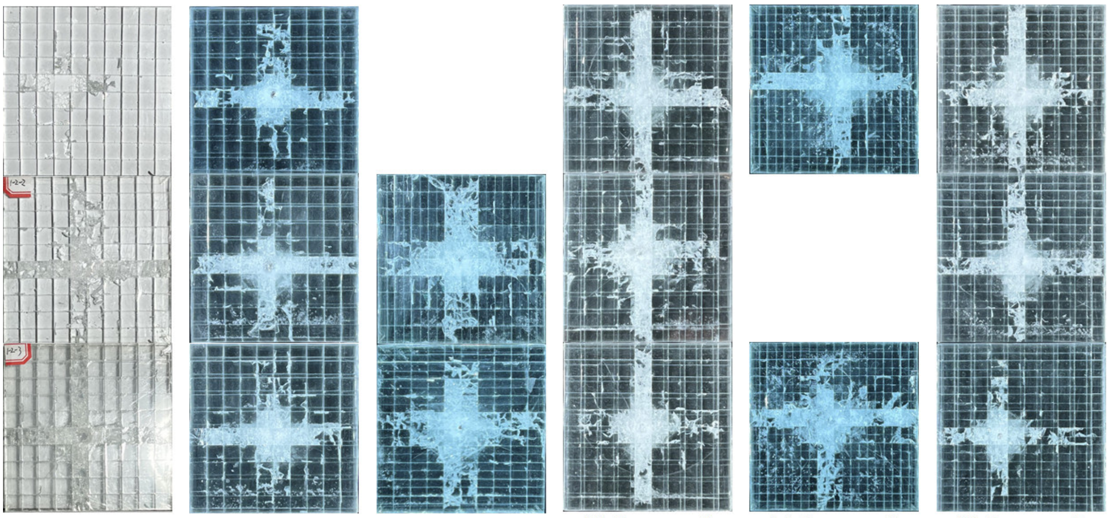


**Fig. S3. Damage patterns for various tablet arrangements of the nacreous structure.**

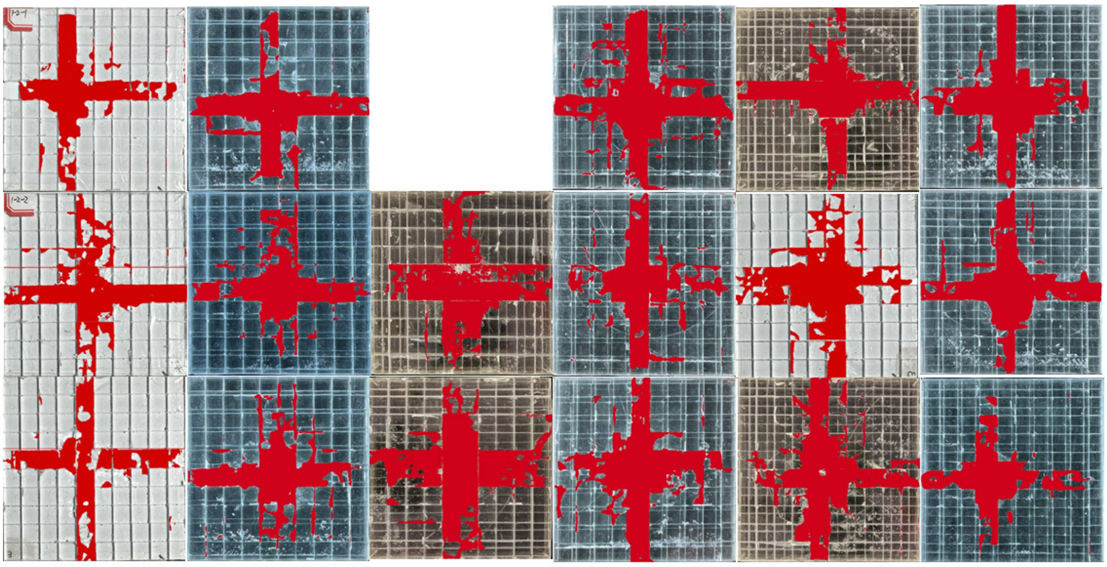


**Fig. S4. Damage areas calculated by Artificial Intelligence for the nacreous transparent composites with various tablets' arrangements.**

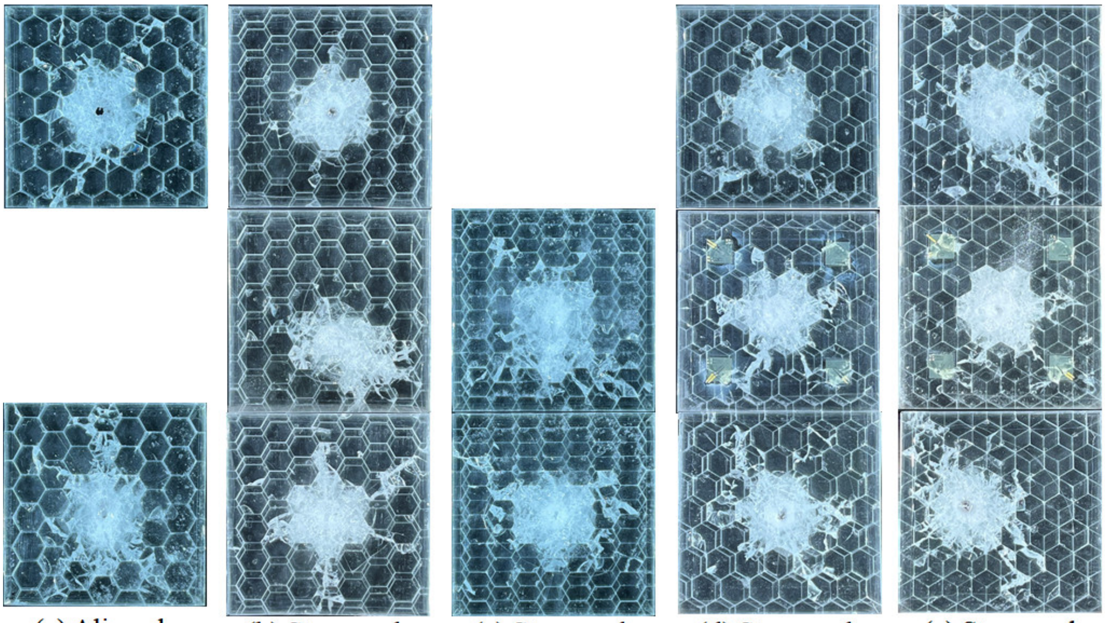


**Fig. S5. Damage patterns for the tortoiseshell structures.**

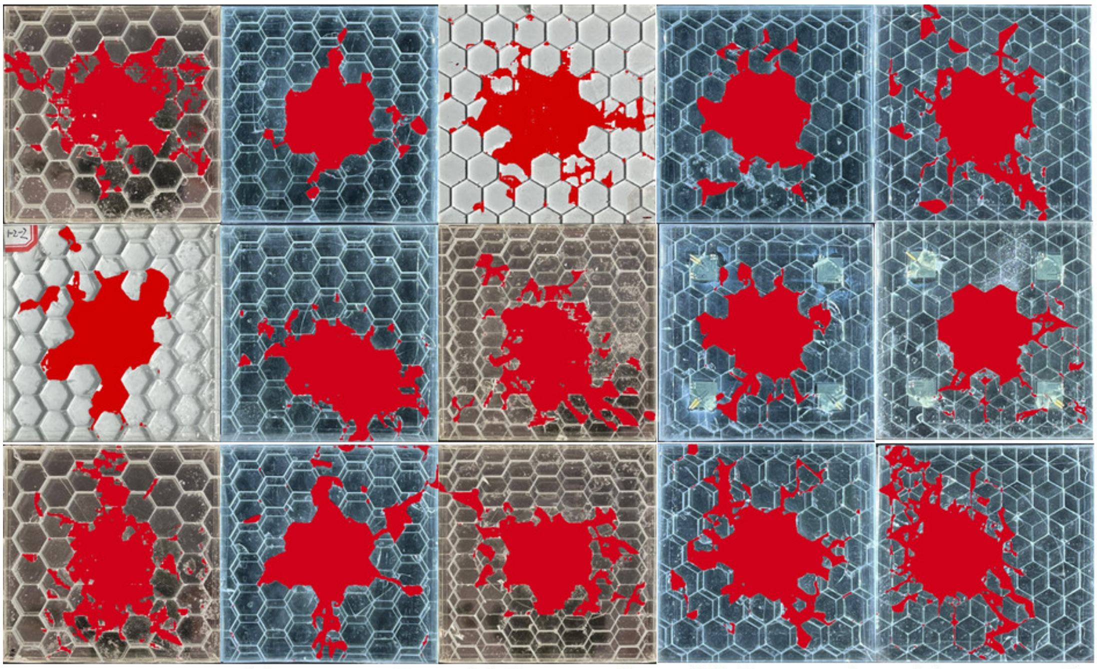


**Fig. S6. Damage areas calculated by Artificial Intelligence for the tortoiseshell-structured composites with various tablets' arrangements.**

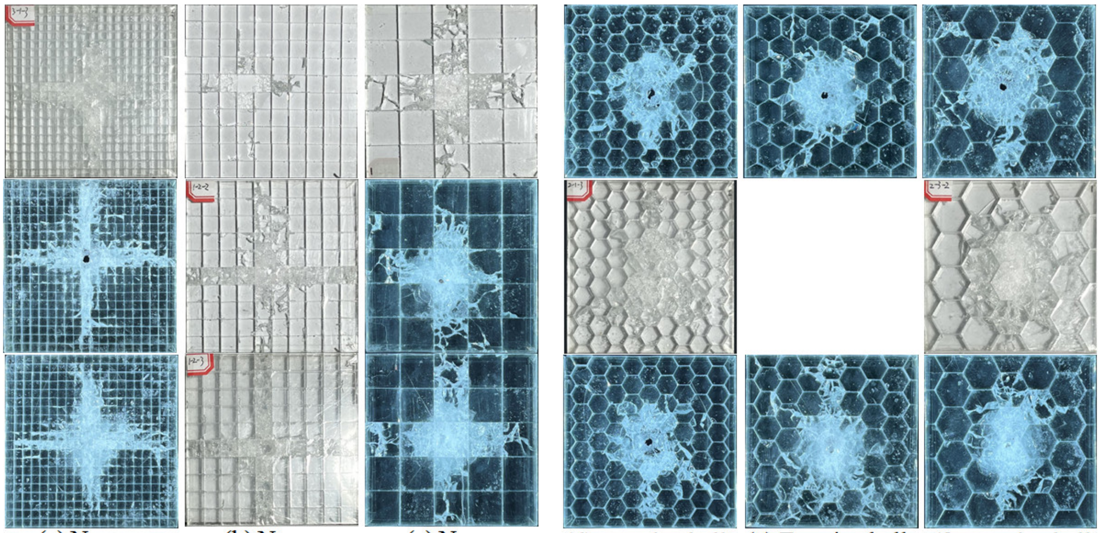

(a) Nacre, Small tablet | (b) Nacre, Medium tablet | (c) Nacre, Large tablet | (d) Tortoiseshell, Small tablet | (e) Tortoiseshell, Medium tablet | (f) Tortoiseshell, Large tablet

**Fig. S7. Damage patterns for biomimetic structures of various tablet sizes.**

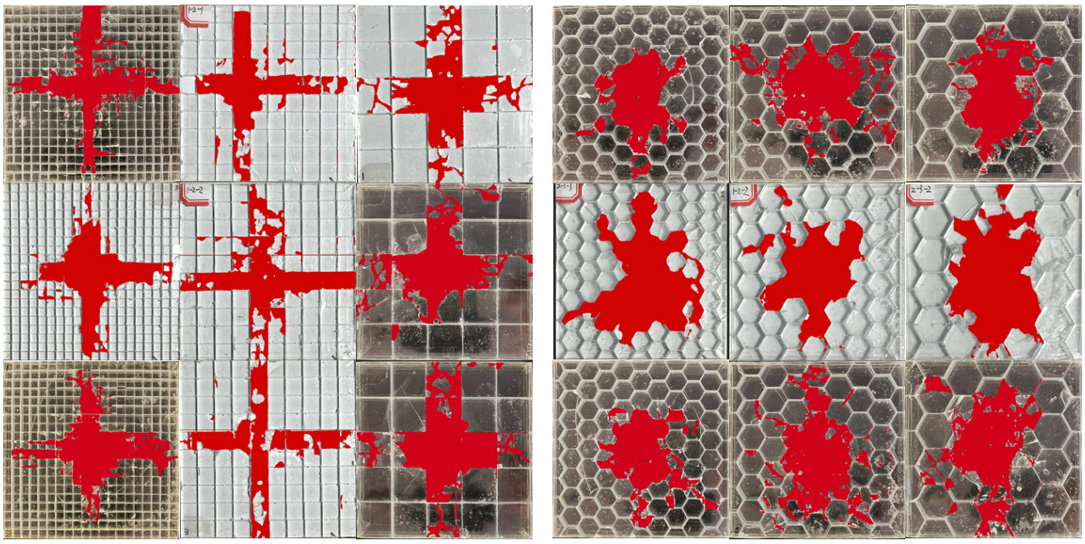

(a) Nacre, Small tablet | (b) Nacre, Medium tablet | (c) Nacre, Large tablet | (d) Tortoiseshell, Small tablet | (e) Tortoiseshell, Medium tablet | (f) Tortoiseshell, Large tablet

**Fig. S8. Damage areas calculated by Artificial Intelligence for various sizes of biomimetic structures.**
